# High Dietary Folic Acid Supplementation Reduced the Composition of Fatty Acids and Amino Acids in Fortified Eggs

**DOI:** 10.3390/foods13071048

**Published:** 2024-03-29

**Authors:** Ao-Chuan Yu, Yu-Han Deng, Cheng Long, Xi-Hui Sheng, Xiang-Guo Wang, Long-Fei Xiao, Xue-Ze Lv, Xiang-Ning Chen, Li Chen, Xiao-Long Qi

**Affiliations:** 1 Animal Science and Technology College, Beijing University of Agriculture, Beijing 102206, China; yac88@88.com (A.-C.Y.); m18611700491_2@163.com (Y.-H.D.); cheng.long@bua.edu.cn (C.L.); shengxh03@163.com (X.-H.S.); xiangguo731@163.com (X.-G.W.); xiaolf1989@bua.edu.cn (L.-F.X.); 2 Department of Livestock and Poultry Products Testing, Beijing General Station of Animal Husbandry, Beijing 100107, China; lvxueze0310@163.com; 3 Food Science and Engineering College, Beijing University of Agriculture, Beijing 102206, China; cxn@bua.edu.cn; 4 Key Laboratory of Agricultural Product Processing and Quality Control (Co-construction by Ministry and Province), Ministry of Agriculture and Rural Affairs, Beijing 102206, China

**Keywords:** FA, egg yolks, nutrient, nutrient-fortified

## Abstract

Aims: The study aimed to evaluate the effects of dietary folic acid (FA) on the production performance of laying hens, egg quality, and the nutritional differences between eggs fortified with FA and ordinary eggs. Methods: A total of 288 26-week-old Hy-Line Brown laying hens (initial body weights 1.65 ± 0.10 kg) with a similar weight and genetic background were used. A completely randomized design divided the birds into a control group and three treatment groups. Each group consisted of six replicates, with twelve chickens per replicate. Initially, all birds were fed a basal diet for 1 week. Subsequently, they were fed a basal diet supplemented with 0, 5, 10, or 15 mg/kg FA in a premix for a duration of 6 weeks. Results: Supplementation of FA could significantly (*p* < 0.05) enhance the FA content in egg yolks, particularly when 10 mg/kg was used, as it had the most effective enrichment effect. Compared to the control group, the Glu content in the 10 and 15 mg/kg FA groups showed a significant (*p* < 0.05) decrease. Additionally, the contents of Asp, Ile, Tyr, Phe, Cys, and Met in the 15 mg/kg FA group were significantly (*p* < 0.05) lower compared to the other groups. Adding FA did not have significant effects on the levels of vitamin A and vitamin E in egg yolk, but the vitamin D content in the 5 and 10 mg/kg FA groups showed a significant (*p* < 0.05) increase. Furthermore, the addition of FA did not have a significant effect on the levels of Cu, Fe, Mn, Se, and Zn in egg yolk. The dietary FA did not have a significant effect on the total saturated fatty acids (SFA) and polyunsaturated fatty acid (PUFA) content in egg yolk. However, the total monounsaturated fatty acid (MUFA) content in the 5 and 10 mg/kg groups significantly (*p* < 0.05) increased. These changes in nutritional content might be attributed to the increased very low-density lipoprotein (VLDL) protein content. The significant decrease in solute carrier family 1 Member 1 (SLC1A1), solute carrier family 1 Member 2 (SLC1A2), and solute carrier family 1 Member 3 (SLC1A3) gene expression compared to the control group appeared to be the reason for the decrease in amino acid content in egg yolk within the dietary FA group. Conclusion: The findings suggest that the appropriate addition of FA can enhance the levels of MUFA and vitamin D in egg yolks, thereby improving their nutritional value. Excessive intake of FA can decrease the effectiveness of enriching FA in egg yolk and impact the enrichment of certain amino acids. The yolk of eggs produced by adding 10 mg/kg of FA to the feed contains the optimal amount of nutrients. This study informs consumers purchasing FA-fortified eggs.

## 1. Introduction

Eggs are widely recognized as an essential source of nutrients and have garnered significant attention due to their abundant nutritional value, versatile consumption options, and affordable price [1]. They serve as a high quality complementary food for infants and play a crucial role in brain and immune development [2,3]. In recent years, there has been a growing interest among consumers in functional eggs, which possess the ability to lower blood lipids [4], reduce inflammation [5], and enhance immunity [6].

It has been reported that folic acid (FA) supplementation during early pregnancy can reduce the risk of coronary heart disease in offspring and improve their cognitive development [7,8]. Furthermore, FA supplementation has shown potential benefits in improving male erectile dysfunction in reproductive health [9]. Folate deficiency is a widespread issue worldwide, primarily caused by insufficient FA intake [10]. Research has indicated that folate deficiency in humans can result in chromosomal damage, potentially leading to cancer [11]. Since the human body cannot produce FA on its own, external supplementation through food and intestinal microflora is necessary [12]. The importance of supplementing FA is evident. To ensure a stable supplementation of FA, researchers have initiated studies on nutrition-fortified eggs enriched with FA. Studies have demonstrated the feasibility of enriching eggs with FA by incorporating it into the diet [13]. FA-fortified eggs exhibit remarkable stability, with minimal loss even after prolonged storage and cooking [14]. Nutritionally, fortified eggs are produced by adding specific nutrients to eggs, thereby supplementing people’s nutritional requirements. Previous studies have demonstrated that enriching laying hen feed with palm oil and algal astaxanthin can increase the levels of tocotrienols, tocopherols, and astaxanthin in egg yolks [15]. Similarly, the addition of corn gluten meal and alfalfa to the feed can enhance the lutein content in egg yolks [16]. Lu et al. successfully increased the selenium content in eggs by incorporating selenium-rich yeast powder into the feed [17]. However, most studies have primarily focused on nutrient enrichment in eggs, disregarding the original nutrient composition of eggs. The purpose of this study is to investigate the impact of dietary FA on the intrinsic nutrients of eggs and its mechanism of action. The assumption is made that the addition of FA will not alter the intrinsic nutrients of eggs, but rather enhance the FA content in eggs.

## 2. Materials and Methods

### 2.1. Animal Care and Use

The Beijing University of Agriculture’s Animal Care and Use Committee approved all experimental protocols (Approval ID: BUA-zc-20200073).

### 2.2. Experimental Materials

FA was purchased from Guangdong Osman Biotechnology Co., Ltd. (Guangzhou, China), with a content of 99%.

### 2.3. Birds and Diets

A total of 288 26-week-old Hy-Line Brown laying hens (initial body weights 1.65 ± 0.10 kg) with a similar weight and genetic background were used. A completely randomized design divided the birds into a control group and three treatment groups. Each group consisted of 6 replicates, with 12 chickens per replicate. Initially, all birds were fed a basal diet for 1 week. Subsequently, they were fed a basal diet supplemented with 0, 5, 10, or 15 mg/kg FA in a premix for a duration of 6 weeks. Table 1 is formulated based on the main nutritional indicators of laying hen compound feeds in formula feeds for layers and broilers (GB/T 5916-2020) [18]. Experimental laying hens were housed in three-tier semi-open three-dimensional cages, with three chickens in each cage. The feeding period lasted for 6 weeks, with a photoperiod of 16 h. The hens were fed twice a day at 7:00 and 16:00, and eggs were collected once daily at 16:30. The hens had unrestricted access to food and water, and the cages were disinfected weekly. The experimental chickens received regular vaccinations.

### 2.4. Sample Collection

The number of eggs as well as the egg weight were recorded daily. Egg production was expressed as average hen day production, calculated from the total eggs divided by the total number of hen days. The average egg weight was calculated as total egg weight divided by the number of eggs. Feed consumption was recorded on a replicate basis at weekly intervals. The feed conversion ratio was recorded as a kilogram of feed consumed per kilogram of eggs produced. Egg quality was measured from 5 eggs randomly collected from each replicate on the 42nd days. After a 6-week feeding trial, the FA contents reached a steady state in the egg yolks. Two hundred and sixteen undamaged eggs were collected and the yolks were separated to prepare the dry yolk powder; the egg yolk was freeze-dried using a lyophilizer (NAI; Shanghai, China). The egg yolk was ground and placed in a bag, which was sealed and stored in a refrigerator at −80 °C. An aliquot was taken for analysis of the FA concentration, amino acid distribution, fatty acid distribution, and vitamin and mineral content. At the end of the feeding period, one healthy bird from each replicate was randomly selected (1 bird per replicate, and 24 birds in total). Immediately upon sacrifice, blood samples were collected via exsanguination of the left jugular vein with scalpels and centrifuged at 4 °C at 4000× *g* for 10 min to separate plasma. Then the plasma samples were frozen at −80 °C until analysis. Then birds were euthanized by exsanguination and necropsied, and the liver and uterus were immediately separated, and quickly frozen at −80 °C for further analysis.

### 2.5. Performance and Egg Quality Measurement

The egg production rate was calculated as follows: (1) The feed efficiency was calculated weekly. An egg analyzer (Orka Food Technology Ltd., Ramat Hasharon, Israel) was used to measure the Haugh unit, the yolk color, and the albumen height. The yolk color was defined according to the Roche yolk color fan, where 1 represents bright yellow and 15 represents dark yellow. An egg force reader (Herzliya, Tel Aviv, Israel) was used to measure the eggshell strength. Finally, the eggshell thickness (ST) was estimated using the following formula (2), where SW = eggshell weight, ES = egg surface area, and d = material density (2.3 g/cm^3^ for calcium carbonate).

(1) $$Egg\;production\;rate=\frac{Number\;of\;eggs}{Total\;birds\times Days}$$

(2) $$ST=\frac{SW}{ES\times d}$$

### 2.6. Determination of Nutrients in the Egg Yolk

In this study, we conducted an analysis of the nutrients in egg yolk powder. Samples were collected from each set of replicates and subjected to analysis in three parallel runs. The determination of FA in egg yolk follows the national standard GB 5009.211-2014 ‘National Food Safety Standard for Determination of FA in Foods’ [19]. FA is a vital nutrient for the growth of *Lactobacillus rhamnosus* (ATCC 7469). In a controlled environment, the lactobacilli liquid is introduced into a culture medium containing the sample liquid. After a specific incubation period, the light transmittance (or absorbance value) is measured. The concentration of FA in the sample is then calculated using the standard curve of FA content and light transmittance (or absorbance value).

The determination of individual amino acids in the egg yolks was carried out following the method described by Li et al [20]. Single amino acids were detected using an amino acid automatic analyzer (model: Biochrom 30, Biochrom Ltd., Cambridge, UK) at a wavelength range of 440 nm to 570 nm. The amino acids detected included aspartic acid (Asp), threonine (Thr), serine (Ser), glutamic acid (Glu), glycine (Gly), alanine (Ala), valine (Val), methionine (Met), isoleucine (Ile), leucine (Leu), tyrosine (Tyr), phenylalanine (Phe), histidine (His), lysine (Lys), arginine (Arg), and proline (Pro), making a total of 18 amino acids. The detection signals were measured at UV 338 nm and fluorescence (EX = 266 nm, EM = 305 nm). The mobile phase A consisted of 40 mmol/L sodium dihydrogen phosphate (pH = 7.8), while the mobile phase B consisted of acetonitrile (45%), methanol (45%), and water (10%).

The determination of individual fatty acids in the egg yolks was carried out following the method described by Luo et al [21]. To characterize the chromatographic peaks, a single standard solution and a mixed standard solution of fatty acid methyl esters were injected into a gas chromatograph (GC). Individual fatty acids were analyzed using a gas chromatography model (Agilent Technologies, Santa Clara, CA, USA). The gas chromatography conditions were as follows: a polydicyanopropylsiloxane strong polar stationary phase capillary column (100 m × 0.25 mm × 0.2 μm) was used. The injection temperature was set at 270 °C, while the detector temperature was 280 °C. The temperature program started at 100 °C and lasted for 13 min, then increased to 180 °C at a rate of 10 °C/min and continued for 6 min. Subsequently, the temperature was increased to 200 °C at a rate of 1 °C/min and held for 20 min, followed by a further increase to 230°C at a rate of 4 °C/min for 10.5 min. Nitrogen was used as the carrier gas, with a split ratio of 100:1, and an injection volume of 1.0 μL. The determination employed the peak area normalization method.

The concentrations of copper (Cu), iron (Fe), zinc (Zn), selenium (Se), and manganese (Mn) in egg yolks were analyzed using inductively coupled plasma atomic emission spectrometry (ICP-AES) with an Optima 8300 instrument (Perkin Elmer, Waltham, MA, USA), following Chen’s method [22].

The concentrations of VTG and VLDL in the egg yolk, plasma, liver, and ovary were measured using commercially available ELISA kits specifically designed for chickens (Nanjing Jiancheng Bioengineering Institute, Nanjing, China), following the instructions provided.

### 2.7. Quantitative PCR Analysis

Total RNA was extracted from 0.1 g of liver and uterus tissue sample using TRIzol reagent (Thermo Fisher Scientific, Shanghai, China), as per the manufacturer’s instructions, and then reverse-transcribed into single-stranded cDNA using the Thermo First cDNA Synthesis Kit (Promega, Beijing, China). The gene expressions of very low-density *lipoprotein receptor (VLDLR)*, *folate receptor 1 (FR1)*, *reduced folate carrier (RFC)*, *solute carrier family 1 Member 1 (SLC1A1)*, *solute carrier family 1 Member 2 (SLC1A2)*, and *solute carrier family 1 Member 3 (SLC1A3)*, were determined using qRT-PCR with specific primers (Table 2). Quantitative PCR analyses were performed using the Step One Plus Real-time PCR system (Applied Biosystems, Foster City, CA, USA). After initial denaturation at 95 °C for 10 min, 40 cycles of amplification were carried out (95 °C for 10 s and 58.2 °C for 30 s), followed by the generation of melt curves that could be used to verify the specificity of amplification. The samples were tested in triplicate. The 2^−ΔΔCT^ [23] method was used to calculate the relative gene expression levels. Refer to the operation of Yu [24] for the specific process.

### 2.8. Statistical Analysis

Statistical analysis was carried out with SPSS for the Microsoft Windows 11.0 program. One-way ANOVA, with orthogonal linear and quadratic contrasts followed by Tukey’s multiple comparison test, was used to examine statistical differences among treatments. Statistical significance was defined at *p* < 0.05. Pearson correlation analysis and PCA were performed based on dimensionality reduction (Origin Pro, version 2021. Originlab Corporation, Northampton, MA, USA). Similarity analysis (ANOSIM) was conducted using the Bray-Curtis method to examine the variations in nutrient composition among eggs fortified with different concentrations of FA.

## 3. Results

### 3.1. Performance and Egg Quality

The effects of dietary FA addition on performance and egg quality are shown in Table 3 and Table 4. We found no significant effects of increasing FA supplementation on performance and egg quality.

### 3.2. Nutrients in Egg Yolks

**Figure 1:**
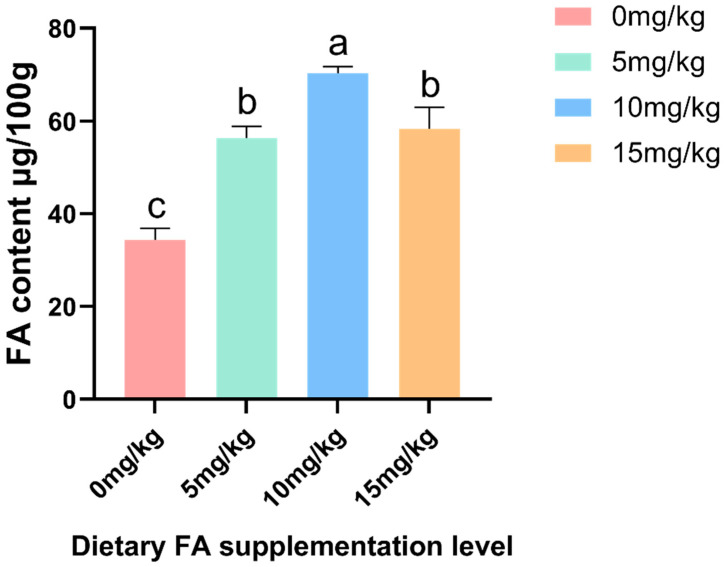
Effects of different levels of dietary FA supplementation on egg yolk FA content (the actual amounts of FA added in the 0, 5, 10, or 15 mg/kg FA supplementation groups were 6, 11, 16, and 21 mg/kg, respectively). ^a–c^ Means within a row with no common superscripts significantly differ (*p* < 0.05).

**Figure 2:**
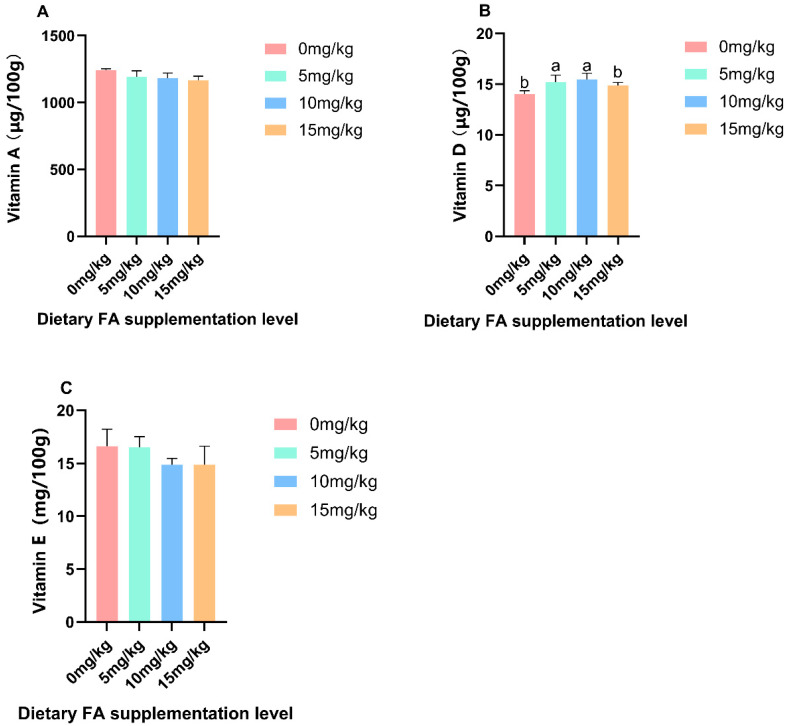
Effect of dietary FA supplementation on vitamins of Egg Yolk. (**A**) Vitamin A; (**B**) Vitamin D; (**C**) Vitamin E. ^a,b^ Means within a row with no common superscripts significantly differ (*p* < 0.05).

**Figure 3:**
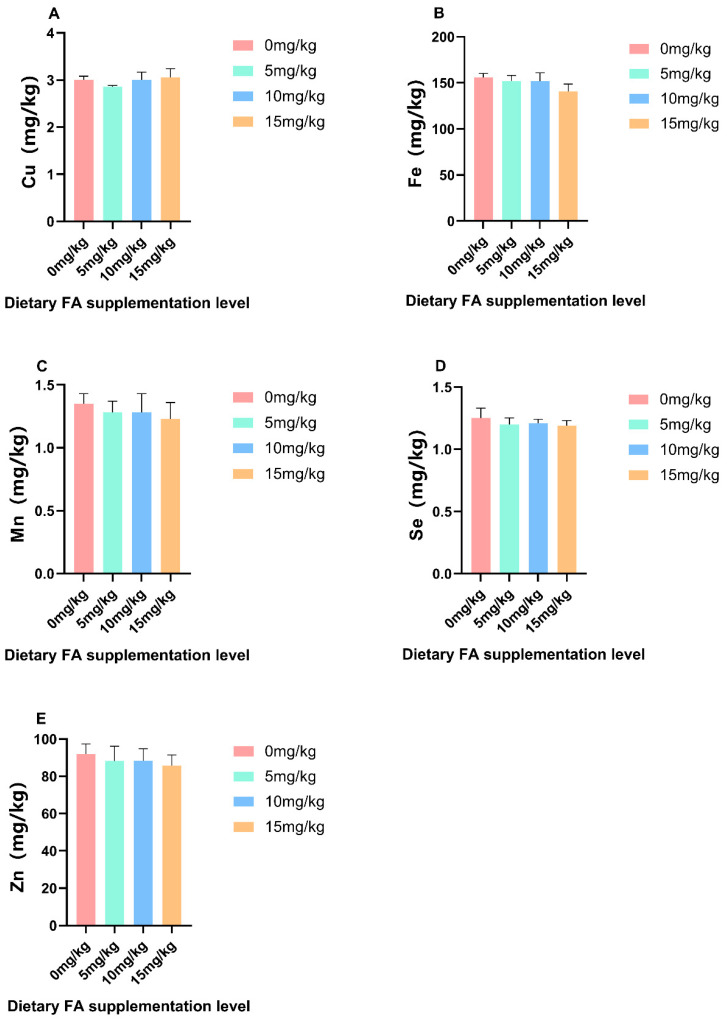
Effect of dietary FA supplementation on trace element of egg yolk. (**A**) copper; (**B**) iron; (**C**) manganese; (**D**) selenium; (**E**) zinc.

**Figure 4:**
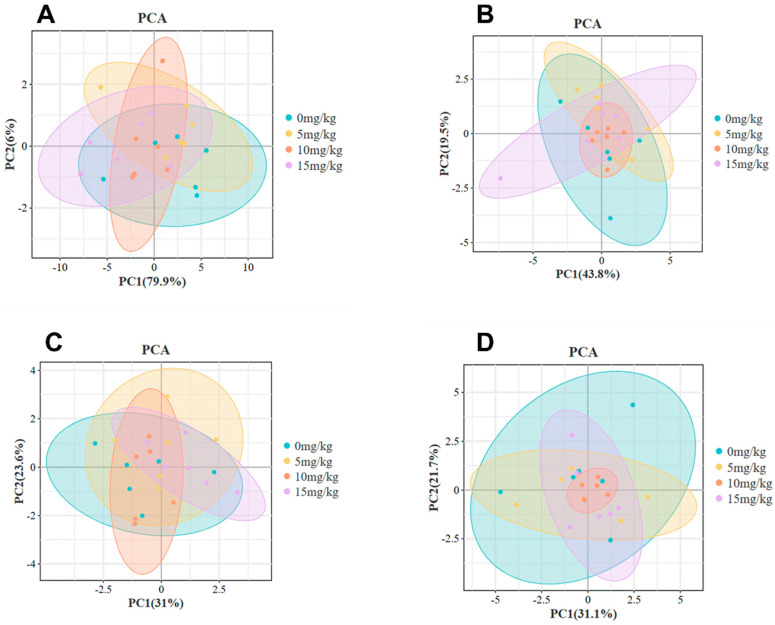
Presentation of the results of PCA analysis for nutrients in egg yolks. (**A**) amino acids; (**B**) SFA; (**C**) MUFA; (**D**) PUFA.

The effect of the dietary addition of FA on nutrients in egg yolks is shown in Table 5 and Table 6 and Figure 1, Figure 2, Figure 3 and Figure 4. The supplementation of FA can significantly (*p* < 0.05) enhance the FA content in egg yolks, particularly when 10 mg/kg is used, as it has the most effective enrichment effect (Figure 1). As the addition of FA in feed increases, there are quadratic effects observed in the levels of FA in egg yolk. Compared to the control group, the Glu content in the 10 and 15 mg/kg FA groups showed a significant (*p* < 0.05) decrease. Additionally, the contents of Asp, Ile, Tyr, Phe, Cys, and Met in the 15 mg/kg FA group were significantly (*p* < 0.05) lower compared to the other groups. Simultaneously, the inclusion of FA in the feed led to a linear decrease in Asp, Glu, Tyr, and Phe levels in the egg yolk. The PCA analysis revealed a notable (*p* = 0.048) disparity in the overall amino acid content among the FA groups that were fed 5 and 15 mg/kg (Figure 4A). When FA was added, there was no significant difference in the total content of saturated fatty acid (SFA) and polyunsaturated fatty acid (PUFA) compared to the control group. However, the total MUFA content in the 5 and 10 mg/kg FA groups showed a significant (*p* < 0.05) increase. As the addition of FA in feed increased, there were quadratic effects observed in the levels of C8:0, C18:1n9t, and MUFA in the egg yolk. Similar results were observed in the PCA analysis, indicating significant variations in the total amount of MUFA between the groups that were fed 5 (*p* = 0.003) and 10 (*p* = 0.008) mg/kg FA, in comparison to the control group (Figure 4C). Furthermore, the C14:1 content in the 5 and 15 mg/kg FA groups was significantly reduced (*p* < 0.05), while the C18:1n9t content in the 5 and 10 mg/kg FA groups was significantly (*p* < 0.05) increased. The group supplemented with 5 mg/kg FA exhibited a significantly (*p* < 0.05) higher C8:0 content compared to the other groups, and the C18:2n6c content was significantly (*p* < 0.05) lower than that of the other groups. Adding FA did not have a significant effect on the levels of vitamin A and vitamin E in the egg yolk, but the vitamin D content in the 5 and 10 mg/kg FA groups showed a significant (*p* < 0.05) increase. As the addition of FA in feed increased, there were quadratic effects observed in the levels of vitamin D in the egg yolk. Furthermore, the addition of FA did not have a significant effect on the levels of Cu, Fe, Mn, Se, and Zn in the egg yolk.

### 3.3. Determination of VLDL and VTG Content in Egg Yolk, Plasma, Liver, and Ovaries

**Figure 5:**
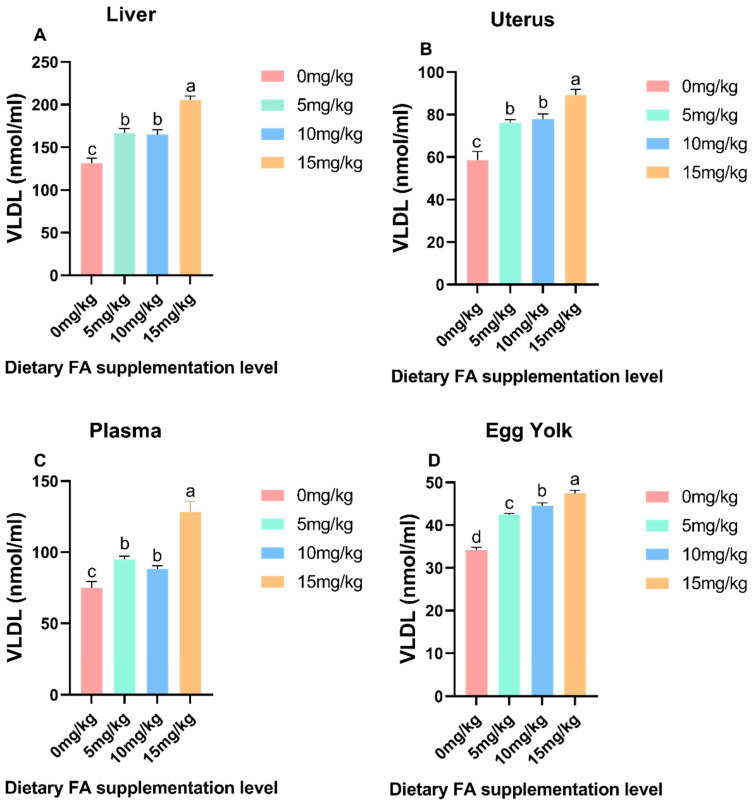
Effect of dietary FA supplementation on the VLDL content in the egg yolk, plasma, liver, and uterus of laying hens. (**A**) Liver; (**B**) Uterus; (**C**) Plasma; (**D**) Egg Yolk. Values are expressed as means ± SD of six birds per treatment. Means without a common letter differ (*p* < 0.05).

**Figure 6:**
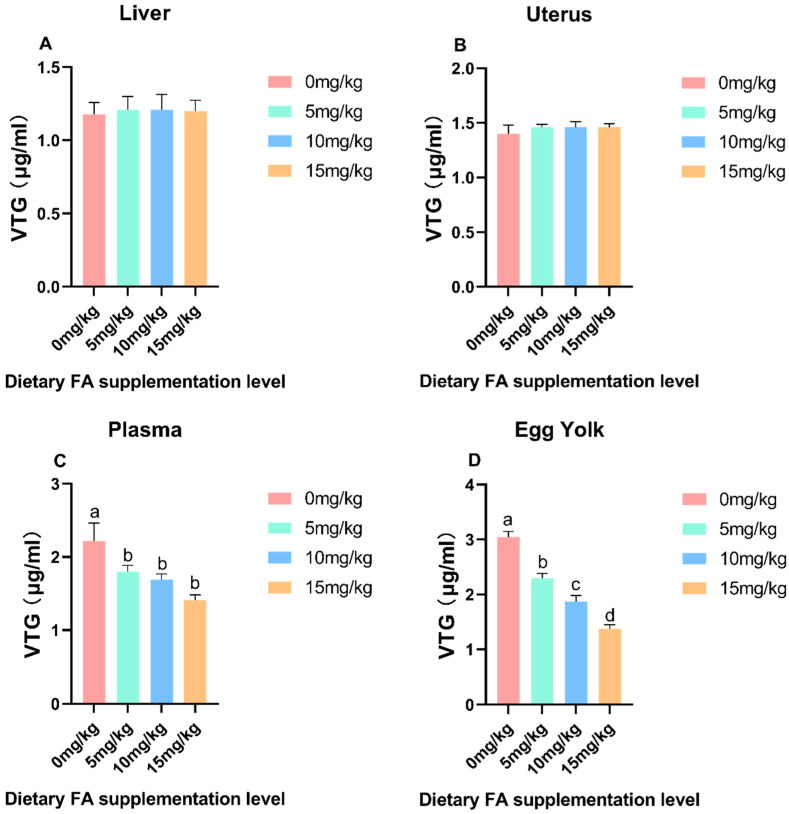
Effect of dietary FA supplementation on the VTG content in egg yolk, plasma, liver, and uterus of laying hens. (**A**) Liver; (**B**) Uterus; (**C**) Plasma; (**D**) Egg Yolk. Values are expressed as means ± SD of six birds per treatment. Means without a common letter differ (*p* < 0.05).

The effect of dietary FA supplementation on the VLDL and VTG content in the egg yolk, plasma, liver, and ovaries are depicted in Figure 5 and Figure 6. Compared to the control group, the addition of FA significantly increased (*p* < 0.05) the VLDL content in the liver, uterus, plasma, and egg yolk. However, the addition of FA had no significant effect on the VTG content in the liver and uterus. Interestingly, it did significantly (*p* < 0.05) reduce the VTG content in plasma and egg yolk. In the egg yolk and plasma, a significant (*p* < 0.05) increase in the protein level of VTG was noticed in the FA supplementation compared to the control.

### 3.4. Mechanism of the Effect of FA on Nutrients in Egg Yolks

**Figure 7:**
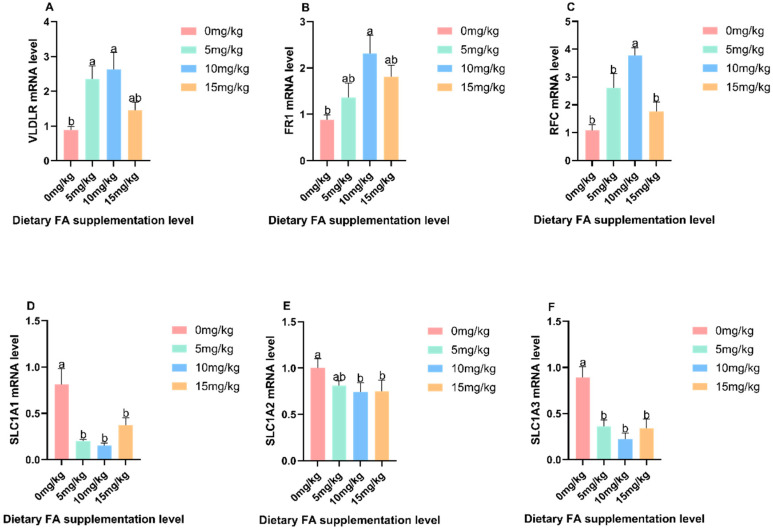
Effects of dietary FA supplementation on nutrient enrichment mRNA expression in the liver: (**A**) *VLDLR*, (**B**) *FR1*, (**C**) *RFC*, (**D**) *SLC1A1*, (**E**) *SLC1A12*, and (**F**) *SLC1A3*. Values are expressed as means ± SD of six birds per treatment. Means without a common letter differ (*p* < 0.05).

**Figure 8:**
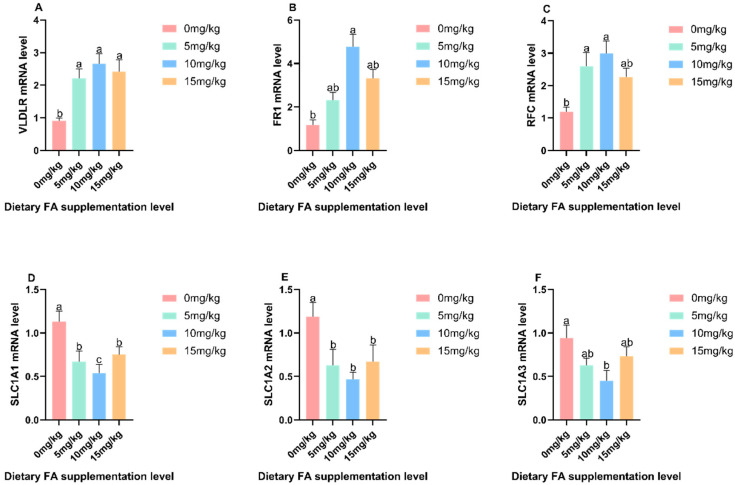
Effects of dietary FA supplementation on nutrient enrichment mRNA expression in the uterus: (**A**) *VLDLR*, (**B**) *FR1*, (**C**) *RFC*, (**D**) *SLC1A1*, (**E**) *SLC1A12*, and (**F**) *SLC1A3*. Values are expressed as means ± SD of six birds per treatment. Means without a common letter differ (*p* < 0.05).

The effects of dietary FA supplementation on the mRNA expression of *VLDLR*, *FR1*, *RFC*, *SLC1A1*, *SLC1A2*, and *SLC1A3* in the liver and uterus are depicted in Figure 7 and Figure 8, respectively. In the liver, VLDLR was significantly (*p* < 0.05) increased in the 5 and 10 mg/kg FA groups compared to the control group. Additionally, *FR1* and *RFC* were significantly (*p* < 0.05) increased in the 10 mg/kg FA group. However, *SLC1A1*, *SLC1A2*, and *SLC1A3* were significantly (*p* < 0.05) reduced in the dietary FA group. Moving on to the uterus, the *RFC* of the 5 and 10 mg/kg FA groups showed a significant (*p* < 0.05) increase compared to the control group. Similarly, the *FR1* of the 10 mg/kg FA group also showed a significant (*p* < 0.05) increase, and the *VLDLR* of the dietary FA group displayed a significant (*p* < 0.05) increase. Furthermore, in the uterus, *SLC1A1* and *SLC1A2* were significantly (*p* < 0.05) decreased in the dietary FA group, and *SLC1A3* was significantly (*p* < 0.05) decreased in the 10 mg/kg FA group.

## 4. Discussion

The purpose of this study was to investigate the variations in nutrient content between FA nutritionally fortified eggs and regular eggs, as well as to explore the underlying mechanisms. The results of this study indicate that dietary FA had no significant impact on the production performance and egg quality of laying hens. This was consistent with previous studies [25]. Previous studies have demonstrated that supplementing the diet with FA can enhance the production performance and egg quality of hens during the late laying period [26]. In summary, adding FA to the diet has no adverse effects on the production performance and egg quality of hens during the peak period of egg production, and can also improve the production performance and egg quality of hens in the late egg production period.

Food fortification with micronutrients is a method used to increase the body’s intake of micronutrients [27], thereby protecting the body’s health [28]. Surveys have shown that consumers are willing to consume nutritionally fortified foods [29]. FA, an important nutrient for human growth and development, plays a crucial role in preventing neural tube defects when taken before conception [7]. Additionally, supplementing with FA during pregnancy has been found to benefit children’s neurocognitive development [30]. However, apart from green leafy vegetables, there are limited natural sources of FA in whole foods. Since FA cannot be synthesized by mammals, it must be obtained from external sources such as the diet and intestinal microorganisms [31]. Previous studies have demonstrated the feasibility of producing FA-fortified eggs by adding FA to the feed of laying hens [32]. Furthermore, FA remains stable in eggs, with minimal loss even after storage and cooking [14]. In this study, the successful enrichment of FA in egg yolk was achieved by adding FA to the feed, with the best effect observed at a concentration of 10 mg/kg. The concentration of FA in the egg yolk of the 15 mg/kg FA group started to decrease, suggesting that the high FA concentration might have hindered its absorption.

FA absorption is primarily mediated by a membrane transporter with micro-molar affinities for folates [33]. RFC is a member of the solute carrier family and plays an important role in the uptake of FA [12]. RFC is the major transport system in cells for folate cofactors and antifolate therapeutics [34]. Studies have shown that inactivation of RFC leads to severe folate deficiency in the body [35]. In our experiment, the expression of RFC and FR1 mRNA was consistent with the enrichment trend of FA in egg yolk. Therefore, the increase and subsequent decrease of FA in egg yolk may be attributed to the inhibition of FA-related transport proteins at high concentrations.

Essential amino acids are those that cannot be synthesized by the human body and must be obtained through diet. The small intestine serves as the primary site for the absorption of dietary amino acids. Amino acid transport in the small intestine is primarily facilitated by amino acid transporters present in intestinal epithelial cells [36]. This study found that dietary FA had no significant effect on the amino acids in egg yolk to a certain extent. However, when dietary FA was added at a concentration of 15 mg/kg, a significant decrease in the amino acids in egg yolk was observed. This could be attributed to the influence of dietary FA on amino acid transport in intestinal epithelial cells and protein synthesis [37]. Based on the research results, we conducted an analysis of the genes SLC1A1, SLC1A2, and SLC1A3, which are responsible for the transportation of different amino acids. *SLC1A1* is responsible for the Na-dependent transport of glutamate, while *SLC1A3* transports aspartate and glutamate, providing energy to cells in the absence of extracellular glutamine [38,39]. Our findings indicated a significant decrease in the gene expression of *SLC1A1*, *SLC1A2*, and *SLC1A3* in the liver and ovary when fed to FA. This decrease in gene expression may explain the impact of FA on the enrichment of certain amino acids in egg yolk.

Lipids play a crucial role in neural development, nerve cell differentiation, and migration, making them essential for the proper functioning of the nervous system [40]. It has been observed that a diet high in SFA can lead to increased neuroinflammation, while a diet rich in MUFA can help reduce neuroinflammation [41]. Extensive dietary research has suggested that humans should avoid the excessive consumption of foods, particularly those containing saturated fats [42]. The consumption of SFA may be more likely to contribute to obesity compared to MUFA and PUFA [43]. In the present study, the impact of dietary FA on SFA and PUFA levels in the egg yolks was not found to be significant. However, the groups supplemented with 5 and 10 mg/kg of FA exhibited a significant increase in MUFA levels. Previous studies have demonstrated that the inclusion of alpha-ketoglutarate in the diet can also result in an augmentation of MUFA levels [44]. The increase in MUFA in egg yolk is mainly attributed to the significant increase in C14:1 and C18:1n9t. Zhou’s research suggests that incorporating fermented feedstuff derived from *citri sarcodactylis fructus* can enhance intestinal digestive enzyme activity, improve nutrient utilization, promote intestinal microbial diversity, and impact intestinal flora metabolism, thereby influencing C14:1 levels in broiler muscle [45]. The yolk of eggs contains a concentrated number of vitamins and trace elements [46]. Studies have shown that certain minerals such as selenium and iodine can be enriched through fortified feed [47]. Similarly, the vitamin content in eggs can be regulated by formulating the diet of hens [48]. In this study, there was no significant difference in the levels of vitamin A, vitamin E, Cu, Fe, Mn, Se, and Zn between the yolks of eggs fortified with FA and the group without fatty acid supplementation. However, the 5 and 10 mg/kg FA supplemented groups showed a significant increase in vitamin D levels. The inclusion of sprouts in the diet has been found to enhance the levels of vitamin D in egg yolks through dietary supplementation [49]. The consumption of folate-fortified eggs can increase the body’s intake of vitamin D. Nutrients in poultry egg yolk are primarily carried by very low-density lipoprotein (VLDL) and vitellogenin (VTG), which are delivered to the oocyte through receptor-mediated endocytosis [50]. These yolk precursors, mainly produced by the liver, mainly consist of lipoproteins, particularly VLDL and VTG [51]. The key components of egg yolk include low-density lipoprotein, yolk particles (primarily composed of lecithin and phosphoprotein), and yolk soluble protein [52]. VLDL and VTG play crucial roles in the yolk formation process [53]. VTG, as a lipoprotein, is responsible for supplying glucose, phosphorus, and fat, as well as binding and transporting metal ions such as calcium, zinc, and iron into the oocyte [48]. In this study, the addition of dietary FA increased the content of VLDL and *VLDLR* gene expression in the liver and uterus. The group receiving 10 mg/kg of FA showed the most significant effect, which could potentially explain the higher levels of vitamin D and MUFA in egg yolks.

In this study, the impact of FA on the levels of nutrients in egg yolk was examined by analyzing its effect on the expression of *RFC*, *VLDLR*, *FR1 SLC1A1*, *SLC1A2*, and *SLC1A3* mRNA in the liver and ovary, as well as the protein content of VLDL. The findings indicated that dietary FA has an impact on the levels of amino acids in egg yolk by suppressing the expression of *SLC1A1*, *SLC1A2*, and *SLC1A3* mRNA. In addition, a diet low in FA can enhance the concentration of FA, vitamin D, and MUFA in egg yolk by upregulating the expression of *RFC*, *FR1*, and *VLDLR* mRNA, as well as increasing the protein content of VLDL. However, high concentrations of FA have the opposite effect, inhibiting the expression of these genes and reducing the concentration of FA, vitamin D, and MUFA in egg yolk. The study determined that the optimal amount of FA to achieve these improvements is 10 mg/kg. Nevertheless, the specific mechanism through which dietary FA supplementation affects individual amino acids and fatty acids needs to be further investigated.

## Figures and Tables

**Table 1:** Composition and nutrient levels of the basal diet (as-fed basis) %.

Ingredients	Content	Nutrient Level	Content
Corn	63.55	Metabolizable energy, MJ·kg^−1^	11.15
Soybean meal	26	Crude protein (%)	16.7
Stone powder	8.8	Calcium (%)	3.38
Calcium hydrogen phosphate	0.96	Available phosphorus (%)	0.31
Salt	0.3	dl-Methionine (%)	0.40
Choline chloride	0.12	l-Lysine (%)	0.79
Phytase	0.02		
DL-met	0.122		
Premix ^a^	0.128		
Total ^b^	100		

^a^ The premix provided the following per kilogram of the diet: VA 12,500 IU, VD_3_ 5250 IU, VE 21.25 mg, VK_3_ 4.375 mg, VB_1_ 2.5 mg, VB_2_ 11.25 mg, VB_6_ 6.25 mg, VB_12_ 3 mg, nicotinic acid 50 mg, D-pantothenic acid 40.75 mg, folic acid 6 mg, biotin 2.375 mg, Fe 87.5 mg, Zn 68 mg, Cu 9.5 mg, Mn 75 mg, I 1.5 mg, and Se 0.3 mg. ^b^ The actual amounts of FA added in the 0, 5, 10, or 15 mg/kg FA supplementation groups were 6, 11, 16, and 21 mg/kg, respectively.

**Table 2:** Target genes and internal reference primers.

Genes	Primer Sequence (5′-3′)	Fragment Length (bp)	Annealing Temperature (°C)
*SLC1A1*	F: AGACATTGGTAGTGGTACGGAGAC	116	58
	R: CAAAGCAGCAACTCCTGTGAT		
*SLC1A2*	F: CCGAAGCAAGTAGAAGTGAGGATG	121	58
	R: CACCCAGGATGACACCAAATACAG		
*SLC1A3*	F: TGGTCATTGTGCTTACATCAGTAGG	108	58
	R: AGACATTGGTAGTGGTACGGAGAC		
*FR1*	F: AGGTGCTGCTGGTGCTGTTG	98	58
	R: GGCTTGGTTTTGTGGTGCTTGG		
*RFC*	F: GCCTGTTCCTCACCCTCTTC	92	58
	R: CTGTCCATCTTGTCCCCACC		
*VTG II*	F: GTCGACGGAAGGGTGAGAAG	95	58
	R: GGTCCTTACGATGCCTGAGC		
*VLDLR*	F: TGAGGATGGGTCTGACGAGAG	101	58
	R: CACACTCCAACTCATCAC		
*β-actin*	F: GCCAACAGAGAGAAGATGACAC	118	58
	R: GTAACACCATCACCAGAGTCCA		

**Table 3:** Effect of dietary FA supplementation on the production performance of laying hens.

Item	Time (Week)	FA (mg/kg)				SEM	*p*-Value		
		0	5	10	15		ANOVA	Linear	Quadratic
Laying rate (%)	1–2	86.40	86.01	86.21	85.87	0.01	0.72	0.37	0.94
	3–4	86.02	85.63	85.02	85.80	0.01	0.10	0.33	0.05
	5–6	85.43	85.61	86.19	85.02	0.01	0.27	0.72	0.11
	1–6	85.95	85.75	85.80	85.77	0.69	0.59	0.23	0.92
Feed efficiency (kg/kg)	1–2	2.16	2.11	2.07	2.10	0.17	0.39	0.21	0.26
	3–4	2.05	2.09	1.98	2.02	0.03	0.14	0.19	0.88
	5–6	2.02	2.14	2.13	2.07	0.04	0.17	0.47	0.04
	1–6	2.08	2.11	2.06	2.07	0.01	0.29	0.38	0.39
Egg weight (g/egg)	1–2	61.69	61.27	62.11	61.09	0.01	0.31	0.59	0.46
	3–4	61.86	62.34	62.39	61.36	0.03	0.46	0.53	0.16
	5–6	62.43	61.35	61.69	62.17	0.01	0.42	0.84	0.12
	1–6	62.00	61.66	62.07	61.54	0.01	0.40	0.41	0.72

**Table 4:** Effect of dietary FA supplementation on egg quality.

Item	Time (Week)	FA (mg/kg)				SEM	*p*-Value		
		0	5	10	15		ANOVA	Linear	Quadratic
Haugh unit	1–6	83.99	82.86	85.42	83.71	0.79	0.73	0.81	0.86
Yolk color ^1^	1–6	6.27	6.70	6.49	6.10	0.13	0.40	0.55	0.13
Albumen height	1–6	7.15	7.25	7.64	7.35	0.10	0.43	0.32	0.37
Eggshell strength (N/cm^2^)	1–6	39.19	39.27	40.96	40.55	0.70	0.78	0.39	0.87
Shell thickness (μm)	1–6	0.45	0.47	0.47	0.48	0.01	0.24	0.063	0.54
Shape index	1–6	1.24	1.25	1.25	1.24	0.01	0.90	0.63	0.58

^1^ The yolk color is defined according to Roche yolk color fan; colors range from 1 to 15, where 1 represents bright yellow and 15 represents dark yellow.

**Table 5:** Effects of dietary FA supplementation on amino acid content in egg yolk.

Item	FA (mg/kg)				SEM	*p*-Value		
	0	5	10	15		ANOVA	Linear	Quadratic
Asp	3.00 ^a^	2.99 ^a^	2.93 ^ab^	2.90 ^b^	0.01	0.01	0.00	0.65
Thr	1.62	1.62	1.60	1.58	<0.01	0.12	0.03	0.52
Ser	2.64	2.63	2.62	2.58	0.01	0.12	0.03	0.43
Glu	3.92 ^a^	3.91 ^a^	3.77 ^b^	3.75 ^b^	0.02	0.008	0.00	0.79
Pro	1.24	1.24	1.24	1.22	<0.01	0.30	0.17	0.19
Gly	0.97	0.97	0.97	0.96	<0.01	0.27	0.09	0.37
Ala	0.17	0.17	0.16	0.16	<0.01	0.15	0.03	0.56
Val	1.85	1.86	1.84	1.82	<0.01	0.14	0.06	0.23
Ile	1.63 ^ab^	1.64 ^a^	1.60 ^ab^	1.58 ^b^	<0.01	0.03	0.01	0.22
Leu	2.75	2.75	2.73	2.70	<0.01	0.14	0.04	0.31
Tyr	1.39 ^a^	1.39 ^ab^	1.37 ^ab^	1.35 ^b^	<0.01	0.02	0.00	0.55
Phe	1.38 ^a^	1.36 ^ab^	1.34 ^b^	1.33 ^b^	<0.01	0.008	0.00	0.64
His	0.80	0.79	0.79	0.78	<0.01	0.11	0.02	0.65
Lys	2.47	2.47	2.45	2.42	<0.01	0.12	0.03	0.37
Arg	2.29	2.3	2.29	2.25	<0.01	0.14	0.05	0.21
Cys	0.54 ^a^	0.54 ^a^	0.54 ^a^	0.53 ^b^	0.73	0.007	0.34	0.75
Met	0.78 ^a^	0.75 ^ab^	0.77 ^ab^	0.75 ^b^	<0.01	0.03	0.03	0.72
Trp	0.41	0.40	0.39	0.39	<0.01	0.75	0.38	0.57

^a,b^ Means within a row with no common superscripts significantly differ (*p* < 0.05).

**Table 6:** Effect of dietary FA supplementation on fatty acids in egg yolk.

Item	FA (mg/kg)				SEM	*p*-Value		
	0	5	10	15		ANOVA	Linear	Quadratic
C4:0	0.0074	0.0073	0.0076	0.0073	<0.01	0.64	0.93	0.58
C8:0	0.0058 ^b^	0.0067 ^a^	0.0060 ^ab^	0.0059 ^b^	<0.01	<0.01	0.82	0.00
C10:0	0.013	0.013	0.014	0.013	<0.01	0.41	0.65	0.13
C12:0	0.018 ^ab^	0.018 ^ab^	0.018 ^a^	0.017 ^b^	<0.01	0.05	0.11	0.10
C13:0	0.0031	0.0031	0.0030	0.0030	<0.01	0.78	0.34	0.98
C14:0	0.23	0.21	0.22	0.21	<0.01	0.17	0.17	0.43
C15:0	0.032	0.035	0.033	0.031	<0.01	0.70	0.58	0.46
C16:0	16.04	15.36	15.87	15.14	0.20	0.38	0.24	0.96
C17:0	0.064	0.072	0.061	0.070	<0.01	0.35	0.77	0.84
C18:0	10.77	10.75	10.92	10.70	0.16	0.97	0.99	0.76
C14:1	0.073 ^a^	0.062 ^b^	0.073 ^a^	0.061 ^b^	<0.01	0.02	0.11	0.94
C15:1	0.0053	0.0054	0.0055	0.005	<0.01	0.92	0.56	0.79
C16:1	2.76	2.49	2.74	2.42	0.06	0.09	0.13	0.77
C17:1	0.056	0.057	0.057	0.052	<0.01	0.98	0.82	0.76
C18:1n9t	4.58 ^b^	5.9 ^a^	6.21 ^a^	4.56 ^b^	0.32	0.02	0.87	<0.05
C18:1n9c	12.6	13.03	12.61	11.75	0.18	0.08	0.06	0.07
C20:1n9	0.14	0.14	0.14	0.13	<0.01	0.28	0.50	0.11
C18:2n6t	0.59	0.76	0.97	0.75	0.066	0.25	0.24	0.15
C20:2	0.2	0.19	0.2	0.2	0.003	0.35	0.63	0.73
C18:2n6c	5.88 ^a^	4.99 ^b^	5.34 ^ab^	5.61 ^ab^	0.11	0.02	0.57	<0.01
C18:3n6	0.061	0.081	0.098	0.096	0.02	<0.01	<0.01	<0.01
C18:3n3	0.12	0.12	0.12	0.13	0.00	0.1	0.06	0.39
C20:3, n6	0.24	0.25	0.26	0.27	0.01	0.85	0.41	0.77
C20:4 n6	1.18	1.19	1.17	1.18	0.02	0.99	0.96	0.99
C20:3n3	0.045	0.046	0.046	0.045	0.00	0.66	0.66	0.25
C20:5n3 EPA	0.02	0.02	0.03	0.03	0.001	0.31	0.08	0.51
C22:6 n3 DHA	0.34	0.36	0.34	0.35	0.005	0.86	0.86	0.90
SFA	27.18	26.48	27.15	26.21	0.33	0.67	0.47	0.86
MUFA	20.20 ^b^	21.69 ^a^	21.84 ^a^	18.97 ^b^	0.29	<0.01	0.03	<0.01
PUFA	8.70	8.02	8.57	8.65	0.15	0.39	0.78	0.23

^a,b^ Means within a row with no common superscripts significantly differ (*p* < 0.05).

## Data Availability

The original contributions presented in the study are included in the article, further inquiries can be directed to the corresponding authors.
